# Hybrid Repair of Thoraco-Abdominal Aortic Disease with Complex Renal and Hypogastric Anatomy

**DOI:** 10.3390/jcm14217525

**Published:** 2025-10-23

**Authors:** Fabrizio Minelli, Simona Sica, Francesco Sposato, Antonino Marzullo, Laura Rascio, Ottavia Borghese, Giovanni Tinelli, Yamume Tshomba

**Affiliations:** 1 Università Cattolica del Sacro Cuore, 00136 Rome, Italy; fabrizio.minelli@policlinicogemelli.it (F.M.); dott.francescosposato@gmail.com (F.S.); ninimarzullo70@gmail.com (A.M.); laura.rascio01@icatt.it (L.R.); ottavia.borghese@guest.policlinicogemelli.it (O.B.); giovanni.tinelli@policlinicogemelli.it (G.T.); yamume.tshomba@policlinicogemelli.it (Y.T.); 2 Unit of Vascular Surgery, Fondazione Policlinico Universitario A. Gemelli—IRCCS, 00168 Rome, Italy

**Keywords:** thoraco-abdominal aortic disease, aneurysm, dissection, hybrid approach

## Abstract

**Background**: The treatment of thoraco-abdominal aortic aneurysms (TAAAs) and chronic type B aortic dissections (TAAD), is technically challenging. Traditional open surgery repair carries high morbidity and mortality rates, while endovascular repair is limited by anatomical constraints. This study investigates the safety and effectiveness of a hybrid approach in high-risk patients with TAA disease and complex renal and hypogastric anatomy. **Methods**: This was a retrospective single-center study, including all consecutive patients with TAAA and TAAD with complex renal and/or hypogastric artery anatomy treated with a hybrid approach between 2020 and 2024 in a high-volume aortic center. Primary endpoint was technical success. Secondary endpoints were early complications, overall and aortic-related mortality, aortic-related reintervention, the incidence of endoleaks, and the target vessel (TV) patency and TV instability at 30-day and during follow-up. **Results**: During the study period, a total of 92 patients with TAAA or TAAD were treated at our institution. Five high-risk patients (5.4%) with complex renal/hypogastric artery anatomy underwent open renal debranching and hypogastric revascularization followed by staged endovascular repair with custom-made double fenestrated/branched device. Technical success was achieved in all cases with no intra-operative mortality. No spinal cord ischemia or 30-day mortality occurred. Target vessel patency at 30 days was 90%. At a median follow-up of 38 months (IQR 26–49 months), there were no cases of aortic-related death. **Conclusions**: Hybrid repair is a feasible and effective option for managing complex TAAA and TAAD in high-risk patients. Larger studies with longer follow-up are needed to better define the clinical role of this approach.

## 1. Introduction

The operative management of thoraco-abdominal aortic disease, including thoraco-abdominal aortic aneurysms (TAAAs) and chronic type B aortic dissections (TAAD), is technically demanding and associated with significant perioperative morbidity and mortality.

Despite advances in surgical techniques, conventional open surgical repair (OSR) remains a high-risk procedure, with reported mortality rates ranging from 5% to 16% and paraplegia rates between 4% and 11% [1,2,3,4].

The evolution of endovascular technologies and the use of fenestrated and branched endovascular aortic repair (F/BEVAR) have expanded the therapeutic options for these patients, offering less invasive options with reduced mortality and recovery times [5]. However, anatomical requirements pose limitations for wider application of endovascular repair.

The management of renal arteries with unfavorable anatomy—such as small caliber, steep angulation, or early bifurcation—poses a risk of target vessel occlusion and graft instability [6,7]. Additionally, bilateral hypogastric artery stenosis or occlusion increases the risk of pelvic ischemia, buttock claudication, erectile dysfunction, ischemic colitis, and spinal cord injury [8,9].

In this context, hybrid repair, combining open visceral debranching with delayed or simultaneous F/BEVAR, offers a strategic advantage, particularly in patients with complex renal and iliac anatomy.

This study aims to evaluate the safety, feasibility, and mid-term outcomes of hybrid repair in high-risk patients with TAAA/TAAD and complex renal and hypogastric anatomy treated at a high-volume tertiary referral center.

## 2. Materials and Methods

### 2.1. Study Design

This was a single-arm, non-randomized, retrospective single-center study including all consecutive patients treated for TAAA or TAAD and associated complex renal arteries anatomy and/or hypogastric arteries steno-occlusive disease, between January 2020 and December 2024 at Fondazione Policlinico Gemelli IRCSS in Rome, Italy. The Institutional Review Board and ethics committee approval were not required due to the retrospective design of the study. Individual consent for intervention was obtained from all patients. Data privacy was managed according to the National Privacy Act. Study data were collected and anonymously gathered in a single electronic database. The database included pre-operative demographics, risk factors, anatomical features, procedural details, and follow-up outcomes (post-operative clinical events and imaging examinations).

### 2.2. Study Population

High-risk patients (defined by the American Society of Anesthesiologists (ASA) as class 3 or 4) with thoraco-abdominal aortic aneurysm or chronic aortic dissection, with aortic diameter >55 mm or rapid growth (>10 mm/year), were included.

The anatomical criteria for selection to the hybrid endovascular approach included the presence of at least one of the following:

- Complex renal artery anatomy:

Diameter < 5 mm

Bifurcation < 2 cm from the ostium

Angulation > 30°

- Bilateral steno-occlusion of hypogastric arteries
- Infrarenal aortic diameter < 38 mm

The treatment strategy was decided for each case after multidisciplinary discussion. Each case was analyzed, planned, and performed by an experienced operator (>50 aortic procedures/year). Patients with aortic rupture or in an emergency setting, including hemodynamic instability at the time of enrollment, were excluded from the study.

### 2.3. Procedural Details

**Figure 1:**
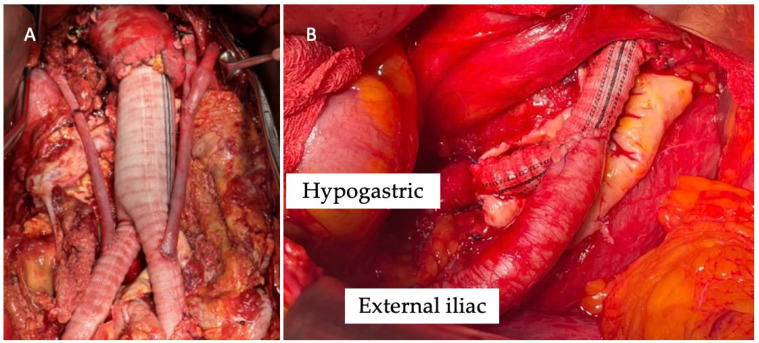
Intra-operative image of renal debranching with great saphenous vein (**A**) and direct revascularization of the hypogastric artery (**B**).

Renal debranching was performed via a midline laparotomy with transperitoneal access. The abdominal aorta was reconstructed with a Dacron graft. Renal arteries were bypassed using PTFE or autologous venous grafts from the iliac arteries. In cases of renal ischemia exceeding 10 min, perfusion was maintained with histidine–tryptophan–ketoglutarate (HTK) solution (Custodiol; Dr. Franz-Kohler Chemie GmbH, Bensheim, Germany). Direct revascularization of the hypogastric artery was performed with a Dacron graft. End-to-end anastomoses were performed with 6/0 polypropylene sutures (Figure 1).

**Figure 2:**
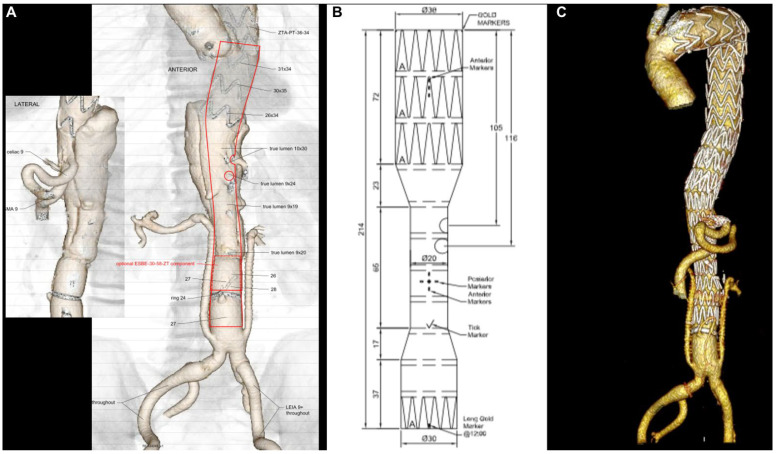
Pre-operative planning of the custom-made device (Cook Medical, Bloomington, Ind) with two fenestrations for the celiac trunk and superior mesenteric artery (**A**,**B**); final 3D volume rendering reconstruction of TAAA hybrid repair with renal debranching, hypogastric revascularization, and endovascular completion using the custom-made device (**C**).

Thoraco-abdominal endovascular exclusion was achieved with the use of thoracic and thoraco-abdominal custom-made devices (CMD) with two fenestrations/branches, tailored to each patient’s anatomy (Figure 2).

The endovascular procedure was performed under general anesthesia, in a hybrid operating room and under fusion imaging guidance. Patients underwent dual antiplatelet therapy for 6 months after the endovascular procedure.

### 2.4. Follow-Up

Patients were observed at regular post-operative appointments. Post-operative surveillance included contrast-enhanced computed tomography angiography (CTA) at 1 month, 6 months, and annually thereafter. Data collected encompassed clinical events, graft patency, and imaging findings. Imaging was evaluated by two independent reviewers.

### 2.5. Endpoints

Outcomes are described in accordance with the current reporting standards [10]. The primary endpoint of this study was the technical success, defined as the correct deployment of the endograft at the intended location, with successful debranching and without intra-operative or immediate post-operative occurrence of surgical conversion or mortality, type I or type III endoleak (EL), graft or target vessel occlusion.

Secondary endpoints were early complications (including spinal cord ischemia (SCI), cardiac, pulmonary, renal complications), the overall mortality and aortic-related mortality, the aortic-related reintervention, the incidence of endoleaks, and the target vessel (TV) patency and TV instability at 30-day and during follow-up. TV instability was defined as any dislocation, stenosis, occlusion, or type Ic/IIIc endoleak involving the visceral branches treated during the procedure.

### 2.6. Statistical Analysis

Quantitative variables were reported as means ± SD or medians (IQR). Categorical variables were presented as counts and percentages. Kaplan–Meier analysis estimated overall survival (OS) and disease-free survival (DFS). Statistical analysis was performed with STATA 15.1 (StataCorp, College Station, TX, USA).

## 3. Results

Between January 2020 and December 2024, a total of 92 patients with TAAA or TAAD were treated at our institution. Five patients (5.4%) with complex renal artery anatomy and/or associated hypogastric artery steno-occlusive were treated with the hybrid approach. The patients’ mean age was 65 years (range 58–71 years). Comorbidities were arterial hypertension in five patients (100%), dyslipidemia in four patients (80%), diabetes in three patients (60%), and coronary artery disease in two patients (40%). Pre-operative therapy was angiotensin-converting enzyme (ACE) inhibitors and mono antiplatelet in five patients (100%), and statin in four cases (80%). Patient’s anatomical details are reported in Table 1.

**Figure 3:**
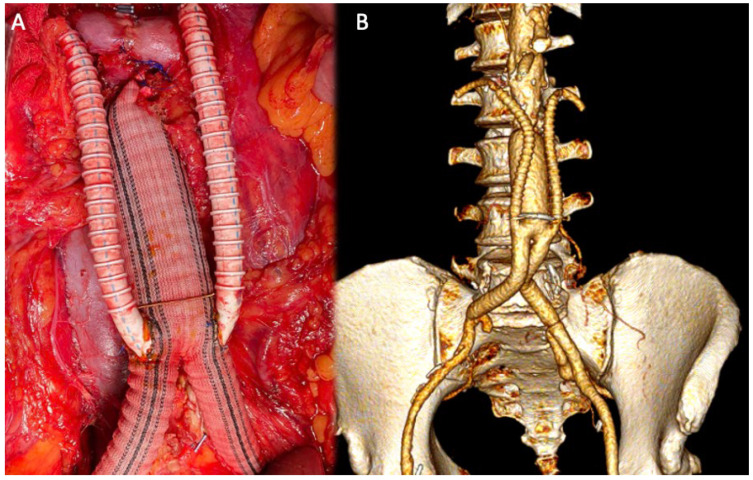
Intra-operative image of renal debranching with PTFE (**A**); 3D volume rendering (VR) reconstruction after debranching (**B**).

The median duration between initial renal debranching and final endovascular exclusion was 109 days (range, 48–210 days). Renal bypass was performed with venous grafts in three cases and PTFE in two patients (Figure 3). In two cases, direct revascularization of the hypogastric arteries was performed using a Dacron graft due to severe bilateral hypogastric artery stenosis.

**Figure 4:**
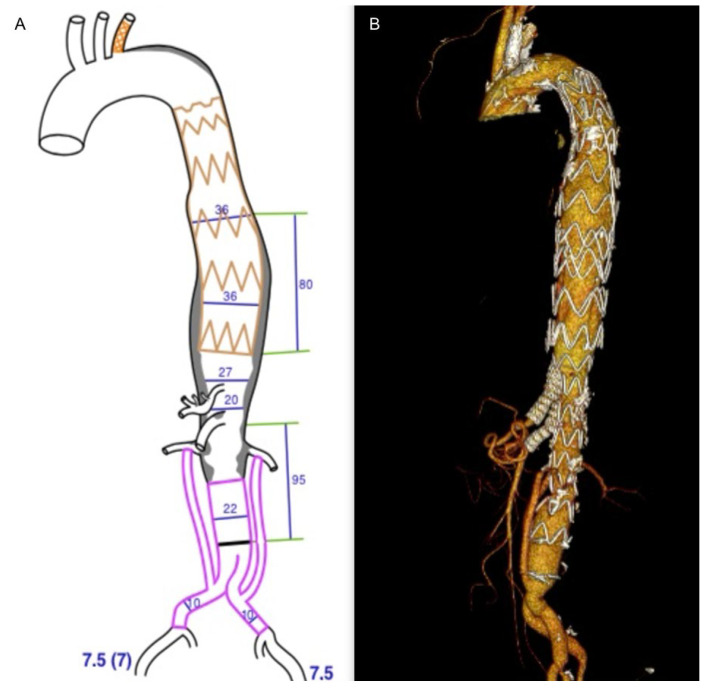
Pre-operative planning of the custom-made device double inner branched endograft (JOTEC, CryoLife, Inc.) (**A**); final 3D volume rendering reconstruction of the hybrid repair (**B**).

All patients subsequently underwent successful implantation of the custom-made thoraco-abdominal device with two fenestrations/branches for the celiac trunk (CT) and superior mesenteric artery (SMA). The CMD used was the double fenestrated graft (Cook Medical, Bloomington, IN, USA) and the double inner branched endograft (JOTEC, CryoLife, Inc., Sarasota, FL, USA) (Figure 4) in four patients and one patient, respectively.

Technical success was achieved in 100% of cases, with successful device deployment without intra-operative mortality and TV/graft-related complications. No intra-operative conversions to open surgery were necessary. No deaths were recorded within 30 days of the procedure. No cases of SCI occurred during hospitalization and at 30 days.

In one patient (Case 5), early occlusion of the right venous renal artery bypass was observed on post-operative day 7. Successful catheter-directed thromboaspiration and balloon-expandable stenting were performed. No other early TV/graft-related complications were observed. The total TV patency rate was 90% (9/10 renal arteries).

Median length of stay after renal revascularization was 11 days (IQR 6–14 days). Subsequent admissions for endovascular completion required a median length of stay of 5 days (IQR 3–7 days).

The median follow-up was 38 months (IQR 26–49 months). All patients completed the intended follow-up period, with no losses reported. No cases of target vessel instability or further TV/graft occlusion were recorded. No deaths occurred during follow-up; no aortic-related reinterventions were performed. At 3-month CTA, there was evidence of one case (Case 4) of type II endoleak, successfully treated with endovascular embolization of the lumbar artery.

Kaplan–Meier analysis revealed that the estimated overall survival was 93% (95% Confidence Interval [CI]: 89–97%) at 1 year, 85% (95% CI: 78–92%) at 3 years, and 78% (95% CI: 68–87%) at 5 years. Freedom from aortic reintervention was 90% at 1 year, 80% at 3 years, and 74% at 5 years.

## 4. Discussion

Over the past decade, less invasive alternatives to OSR for TAAAs and TAADs have become increasingly widespread, aiming to minimize post-operative complications and offer treatment options to patients who are not candidates for conventional surgery [11,12,13]. However, while the endovascular stent-graft has been successfully applied to descending thoracic and abdominal aortic aneurysms, its use in thoraco-abdominal aortic disease is more challenging. Despite the increasing use of fenestrated and branched endovascular aortic repair, a significant proportion of patients remain anatomically unsuitable for this approach. Recent studies have reported that up to 40% of TAAA patients are ineligible for F/BEVAR due to unfavorable anatomical features, such as severe renal artery angulation (>30°), small visceral vessel diameter (<5 mm), short ostial distances, or inadequate landing zones in the true lumen [6,7,10]. These limitations are even more frequent in patients with chronic post-dissection aneurysms, where a higher rate of narrowed true lumens and complex renal or visceral origins are described [7,10,14]. In our experience, 5.2% of TAAAs and TAADs were high-risk patients with complex renal and hypogastric anatomy not suitable for total endovascular repair.

To overcome these limitations, a hybrid technique was introduced, combining open surgical revascularization of the renal and visceral arteries with subsequent endovascular exclusion of the aneurysmal segment [12,15,16,17]. This approach offers several potential benefits in high-risk patients: an adequate distal sealing zone for the endograft, lowering the incidence of type 1B endoleaks; the avoidance of thoracotomy and cardiopulmonary bypass, with faster associated recovery; and a potentially reduced risk of spinal cord ischemia and paraplegia [16].

SCI remains one of the most major complications following complex aortic repair, with reported rates ranging from 4% to 20% depending on the extent of aortic coverage, surgical technique, and patient comorbidities [1,2,3]. The pathophysiology is mainly due to interruption of segmental arterial inflow to the spinal cord, including intercostal, lumbar, and hypogastric arteries [18]. During OSR, especially in extensive Crawford type I or II aneurysms, SCI rates have been reported up to 16%, with permanent deficits in 5–10% of patients despite the use of adjuncts such as cerebrospinal fluid drainage [4,19]. F/BEVAR also present a risk of SCI, particularly in extensive aortic coverage and complex anatomy, with reported rates between 4% and 10% [7,10]. Tshomba et al. reported a 5.6% incidence of SCI in a hybrid series of 52 patients [15]; Quinones-Baldrich et al. found no paraplegia in selected patients undergoing hybrid staged repair with CSF drainage and permissive hypertension [20]. In our experience, no cases of SCI occurred; all our cases were staged. The theoretical benefit of the staged hybrid approach lies in its potential to preserve spinal cord perfusion, allowing collateral remodeling between stages. The staged strategy reduces the burden of the procedure and theoretically reduces the risk of coagulopathy related to the aneurysmal sac thrombosis associated with the extensive surgical procedure required for visceral arteries re-routing [21]. The one-stage strategy offers the advantage of eliminating the risk of rupture between surgeries and provides immediate iliac or aortic access when the femoral arteries are unsuitable [21].

Maintaining long-term stability of target vessels, preventing occlusion, stenosis, or type Ic/IIIc endoleaks are essential for the durability of F/BEVAR. However, TV complications remain a major cause of reintervention. Becker et al. reported that 24% of patients required secondary procedures during follow-up, and 75% of these interventions were related to TV issues [22]. In particular, the variability of renal artery anatomy could influence the outcomes after endovascular TAAA repair. Gallitto et al. reported that upward renal artery orientation and para-visceral aortic angle >45° were associated with renal artery loss (8% of cases) [23]. In case of hybrid repair, the long-term patency and safer route of renal and visceral bypass are matters of concern. Although the hybrid technique can overcome anatomical limitations unsuitable for total endovascular repair, the durability of bypass grafts is critical to prevent late complications such as occlusion or stenosis, which can compromise organ perfusion and patient outcomes. In our cohort, patients presented small, highly angulated, or multiple renal arteries; only one early occlusion of a venous graft used for the left renal artery bypass was reported and promptly managed with thromboaspiration. Careful surgical planning and close post-operative surveillance are essential to maximize graft longevity and minimize the need for reintervention.

Moreover, our series included patients with complex steno-occlusion of the hypogastric arteries; in these cases, direct revascularization was always performed. Hypogastric artery preservation or revascularization is a critical aspect of complex aortic repair, aiming to prevent pelvic ischemic complications such as buttock claudication, erectile dysfunction, and bowel or spinal cord ischemia. Various techniques have been described, including surgical bypass and endovascular approaches. In a study by Wooster et al., retrograde endovascular external-to-internal iliac artery bypass was successfully performed in 17 patients with no ischemic complications reported during a mean follow-up of 30 months [24]. Similarly, open surgical revascularization using Dacron grafts during aneurysm repair has shown durable outcomes with low complication rates [8]. In contrast, a systematic review by Bosanquet et al. found that hypogastric artery occlusion—whether through embolization or coverage—was associated with buttock claudication in 27.9% of patients [9]. In the current study, hypogastric artery preservation or revascularization was carefully considered for each patient. Successful direct revascularizing of the hypogastric artery in two patients using a Dacron graft was performed. Bilateral hypogastric artery occlusion may be tolerated in the presence of collateral pathways, but the risks of ischemic complications remain substantial. Addressing hypogastric artery disease during complex aortic repairs is essential.

Mortality rates following thoraco-abdominal OSR range between 8% and 25%, influenced by aneurysm extent, and surgical complexity requiring thoracotomy and aortic cross-clamping [1,4]. Lower 4.9% mortality rate is reported for F/BEVAR at 30-day; however, the survival at 3 and 5 years is 86.7% and 59.6%, respectively [25]. Factors influencing a higher mortality of complete endovascular aortic repair were high-risk patients’ profile and the need of frequent reintervention [25,26]. Early mortality rates reported after hybrid repair are 5–15%, reflecting a balance between invasiveness and patient complexity [15,27,28,29]. Di Marco et al. reported an overall survival of 75.6% at 3-years in a cohort with hybrid TAAA repair [27]. Our survival analysis is in line with the current literature, showing an overall survival rate of 85% and 78% at 3 and 5 years, respectively.

Hybrid repair in patients with complex renal and hypogastric artery anatomy demonstrates promising outcomes. Our cohort, consisting of patients with challenging anatomical features and high surgical risk, experienced a 100% technical success rate with no early mortality, no SCI, and no TV/graft-related instability.

Despite the promising outcomes, our study is limited by its small sample size, retrospective design, and relatively short follow-up period. Small cohort size may limit the generalizability and statistical power of our findings. Larger prospective studies with longer follow-up are needed to further validate these findings and optimize patient selection criteria for hybrid repair. Additionally, comparative studies with OSR and F/BEVAR will better define the long-term benefits and risks of hybrid repair in this patient population.

## 5. Conclusions

The hybrid approach combining open visceral debranching with custom-made fenestrated and branched endografts provides a safe and effective solution for managing thoraco-abdominal aortic aneurysms and type B aortic dissections in patients with complex renal and hypogastric artery anatomies. The results of this study support the continued use of this technique as a viable alternative to traditional open surgery, particularly in patients with challenging vascular anatomy. Long-term follow-up and larger cohort studies are necessary to confirm the durability of this approach and its place in the management of complex aortic diseases.

## Figures and Tables

**Table 1:** Patient’s details of each patient.

Case	Diagnosis	Renal Arteries Characteristics	Hypogastric Arteries Involvement	Debranching
Case 1 Male	TAAA Crawford Type II	Diameter < 5 mm Angulation > 30°	No	Bilateral renal arteries GSV
Case 2 Male	TAAA Crawford Type II	Short bifurcation < 2 cm	Bilateral occlusion	Bilateral renal arteries PTFE
Case 3 Male	TAAD	Diameter < 5 mm Angulation > 30°	No	Bilateral renal arteries PTFE
Case 4 Female	TAAD	Diameter < 5 mm	No	Bilateral renal arteries GSV
Case 5 Male	TAAD	Multiple renal arteries Angulation > 30°	Bilateral occlusion	Bilateral renal arteries GSV

GSV: great saphenous vein; PTFE: Polytetrafluoroethylene.

## Data Availability

Data is unavailable due to privacy restrictions.

## References

[1] Coselli J.S., Bozinovski J., Lemaire S.A. (2007). Open surgical repair of 2286 thoracoabdominal aortic aneurysms. Ann. Thorac. Surg..

[2] Safi H.J., Estrera A.L., Azizzadeh A., Coogan S., Miller C.C. (2008). Progress and future challenges in thoracoabdominal aortic aneurysm management. World J. Surg..

[3] Cambria R.P., Clouse W.D., Davison J.K., Dunn P.F., Corey M., Dorer D.J. (2002). Thoracoabdominal aneurysm repair: Results with 337 operations performed over a 15-year interval. Ann. Surg..

[4] Estrera A.L., Miller C.C., Chen E.P., Meada R., Torres R.H., Porat E.E., Huynh T.T., Azizzadeh A., Safi H.J. (2005). Descending thoracic aortic aneurysm repair: 12-year experience using distal aortic perfusion and cerebrospinal fluid drainage. Ann. Thorac. Surg..

[5] Oderich G.S., Ribeiro M., Reis de Souza L., Hofer J., Wigham J., Mendes B.C., Vrtiska T. (2017). Endovascular repair of thoracoabdominal aortic aneurysms using fenestrated and branched stent-grafts: Lessons learned. J. Thorac. Cardiovasc. Surg..

[6] Mendes B.C., Oderich G.S., Reis de Souza L., Banga P., Macedo T.A., DeMartino R.R., Misra S., Gloviczki P. (2016). Implications of renal artery anatomy for endovascular repair using fenestrated, branched, or parallel stent graft techniques. J. Vasc. Surg..

[7] Squizzato F., Antonello M., Forcella E., Coppadoro S., Colacchio C., Xodo A., Grego F., Piazza M. (2022). Geometrical determinants of target vessel instability in fenestrated endovascular aortic repair. J. Vasc. Surg..

[8] Sousa L.H.D., Baptista-Silva J.C., Vasconcelos V., Flumignan R.L., Nakano L.C. (2020). Internal iliac artery revascularisation versus internal iliac artery occlusion for endovascular treatment of aorto-iliac aneurysms. Cochrane Database Syst Rev..

[9] Bosanquet D.C., Wilcox C., Whitehurst L., Cox A., Williams I.M., Twine C.P., British Society of Endovascular Therapy (BSET) (2017). Systematic Review and Meta-analysis of the Effect of Internal Iliac Artery Exclusion for Patients Undergoing EVAR. Eur. J. Vasc. Endovasc. Surg..

[10] Oderich G.S., Forbes T.L., Chaer R., Davies M.G., Lindsay T.F., Mastracci T., Singh M.J., Timaran C., Woo E.Y. (2021). Reporting standards for endovascular aortic repair of aneurysms involving the renal-mesenteric arteries. J. Vasc. Surg..

[11] Hughes G.C., Andersen N.D., Hanna J.M., McCann R.L. (2012). Thoracoabdominal aortic aneurysm: Hybrid repair outcomes. Ann. Cardiothorac. Surg..

[12] Pacini D., Di Marco L., Murana G., Pantaleo A., Leone A., Di Bartolomeo R. (2013). Hybrid repair of thoracoabdominal aneurysm: A two-stage approach. Ann. Thorac. Surg..

[13] Patel R., Conrad M.F., Paruchuri V., Kwolek C.J., Chung T.K., Cambria R.P. (2009). Thoracoabdominal aneurysm repair: Hybrid versus open repair. J. Vasc. Surg..

[14] Tinelli G., Lescan M., Sica S., Piazza M., Bisdas T., Makaloski V., Bertoglio L., Verzini F., Pratesi G., Tshomba Y. (2025). Inner Branch Off-the-Shelf Technology for Chronic Post-dissection Thoraco-Abdominal Aneurysm with Narrow True Lumen: Results of a European Multicentre Study. Eur. J. Vasc. Endovasc. Surg..

[15] Tshomba Y., Melissano G., Logaldo D., Rinaldi E., Bertoglio L., Civilini E., Psacharopulo D., Chiesa R. (2012). Clinical outcomes of hybrid repair for thoracoabdominal aortic aneurysms. Ann. Cardiothorac. Surg..

[16] Di Bartolomeo R., Murana G., Cefarelli M., Alfonsi J., Di Marco L., Francesco B., Lovato L., Pacini D. (2016). Hybrid two-stage repair of thoracoabdominal aortic aneurysm. Multimed. Man. Cardiothorac. Surg..

[17] Biasi L., Ali T., Loosemore T., Morgan R., Loftus I., Thompson M. (2009). Hybrid repair of complex thoracoabdominal aortic aneurysms using applied endovascular strategies combined with visceral and renal revascularization. J. Thorac. Cardiovasc. Surg..

[18] Griepp R.B., Di Luozzo G. (2013). Hypothermia for aortic surgery. J. Thorac. Cardiovasc. Surg..

[19] Etz C.D., Halstead J.C., Spielvogel D., Shahani R., Lazala R., Homann T.M., Weisz D.J., Plestis K., Griepp R.B. (2006). Thoracic and thoracoabdominal aneurysm repair: Is reimplantation of intercostal and lumbar arteries a waste of time?. Ann. Thorac. Surg..

[20] Quinones-Baldrich W.J., Jimenez J.C., DeRubertis B.G., Moore W.S. (2009). Combined endovascular and surgical approach (CESA) to thoracoabdominal aortic pathology: A 10-year experience. J. Vasc. Surg..

[21] Chiesa R., Tshomba Y., Melissano G., Logaldo D. (2009). Is Hybrid Procedure the Best Treatment Option for Thoraco-Abdominal Aortic Aneurysm?. Eur. J. Vasc. Endovasc. Surg..

[22] Becker D., Sikman L., Ali A., Prendes C.F., Stana J., Tsilimparis N. (2025). The Impact of Target Vessel Anatomy and Bridging Stent Geometry on Branched Endovascular Aortic Repair Outcomes. Eur. J. Vasc. Endovasc. Surg..

[23] Gallitto E., Faggioli G., Pini R., Mascoli C., Ancetti S., Abualhin M., Stella A., Gargiulo M. (2018). Renal artery orientation influences the renal outcome in endovascular thoraco-abdominal aortic aneurysm repair. Eur. J. Vasc. Endovasc. Surg..

[24] Wooster M., Armstrong P., Back M. (2018). Hypogastric Preservation Using Retrograde Endovascular Bypass. Ann Vasc Surg..

[25] Van Calster K., Bianchini A., Elias F., Hertault A., Azzaoui R., Fabre D., Sobocinski J., Haulon S. (2019). Risk factors for early and late mortality after fenestrated and branched endovascular repair of complex aneurysms. J. Vasc. Surg..

[26] Tinelli G., Sica S., Sobocinski J., Ribreau Z., de Waure C., Ferraresi M., Snider F., Tshomba Y., Haulon S. (2024). Long-term propensity-matched comparison of fenestrated endovascular aneurysm repair and open surgical repair of complex abdominal aortic aneurysms. J. Endovasc. Ther..

[27] Di Marco L., Murana G., Leone A., Alfonsi J., Mariani C., Cefarelli M., Pantaleo A., Pacini D., Di Bartolomeo R. (2018). Hybrid repair of thoracoabdominal aneurysm: An alternative strategy for preventing major complications in high-risk patients. Int. J. Cardiol..

[28] Tshomba Y., Bertoglio L., Marone E.M., Melissano G., Chiesa R. (2008). Visceral aortic patch aneurysm after thoracoabdominal aortic repair: Conventional vs hybrid treatment. J. Vasc. Surg..

[29] Kahlberg A., Tshomba Y., Baccellieri D., Bertoglio L., Rinaldi E., Ardita V., Colombo E., Moscato U., Melissano G., Chiesa R. (2023). Renal perfusion with histidine-tryptophan-ketoglutarate compared with Ringer’s solution in patients undergoing thoracoabdominal aortic open repair. J. Thorac. Cardiovasc. Surg..

